# Late-Onset Bipolar Disorder: Considerations for Diagnosis and Treatment

**DOI:** 10.7759/cureus.39278

**Published:** 2023-05-20

**Authors:** Anna K McKenzie, Rishab Chawla, Bhargav Patel, Reddy B Shashank

**Affiliations:** 1 Department of Neurological Sciences, Rush University Medical Center, Chicago, USA; 2 Department of Psychiatry and Health Behavior, Augusta University Medical College of Georgia, Augusta, USA

**Keywords:** geriatric psychiatry, moca score, electroconvulsive therapy (ect), late-onset, bipolar disorders

## Abstract

Bipolar I disorder is characterized by the presence of at least one manic episode (DSM-5). Despite a decent percentage of individuals being diagnosed later in life, there currently exist no formal treatment guidelines for late-onset bipolar disorder (LOBD), which remains poorly understood. Typically, manic or manic-like episodes in elderly individuals can be thought of as arising from a secondary, physical cause. However, in the absence of a pre-existing neurological disorder - and when laboratory, imaging, and exam findings do not fully support a neurological picture - the determination of a structural versus primary etiology for LOBD becomes challenging.

We present the case of Ms. S, a 79-year-old woman with a past psychiatry history of bipolar disorder diagnosed after 2012 and non-contributory past medical history who was admitted to a state mental hospital on a probate court order from local jail secondary to labile mood and physical aggression toward an officer. Initial labs were remarkable for slightly elevated low-density lipoprotein and a B12 at the lower limit of normal. She was started on a regiment oral B12 supplement, valproic acid 500 mg twice daily, haloperidol 5 mg nightly, and diphenhydramine 25 mg nightly. Despite her medication regimen, she continued to display marked mood lability, tangential thought processes, grandiose delusions, and paranoia. A CT head one week into admission revealed bilateral periventricular white-matter hyperintensities with decreased attenuation and chronic white-matter infarcts. She underwent five sessions of electroconvulsive therapy (ECT), with significantly improving Montreal Cognitive Assessment and Young Mania Rating Scale scores. At the time of discharge on day 32, the patient was fully oriented to self and surroundings with good hygiene, a normal rate of speech, euthymic mood, and congruent affect.

The case of Ms. S underscores the importance of a thorough workup to rule out secondary causes of mania. In addition, it is a clarion call for revisiting and researching a comprehensive management approach to LOBD, for which serial cognitive assessments and ECTs may play an important role.

## Introduction

Bipolar I disorder is characterized by the presence of at least one lifetime manic episode (DSM-5). Its inheritance as a polygenic disease is multifactorial but is considered highly heritable among all psychiatric disorders [1]. Though bipolar I disorder typically manifests in young adulthood, a diagnosis later in life is also common and necessarily involves greater considerations and broad differential diagnosis.

Here, we present the case of Ms. S, a 79-year-old woman with a past psychiatry history of bipolar disorder diagnosed roughly 10 years prior and a non-contributory past medical history who was admitted to a state mental hospital on a probate court order from a local jail secondary to labile mood and physical aggression. The significance of this case is twofold: (1) the average age at diagnosis for bipolar disorder appears to be under 30 years, and (2) the patient’s late-onset bipolar disorder (LOBD) was in the setting of ambiguous objective findings and lack of major predisposing factors [2,3].

Typically, manic or manic-like episodes in elderly individuals can be thought of as arising from a secondary, physical cause (e.g., metabolic, infectious, or drug-induced). Systematic reviews have found that inflammatory processes may mediate the pathogenesis of mania on a molecular level, and potential anti-inflammatory agents for the treatment of depressive symptoms may induce mania [4,5]. Less commonly, mania may even arise as a future sequela of singular cerebrovascular events. Arai et al. presented the case of a 70-year-old female who developed manic symptoms following a cerebral infarction and in the setting of pre-existing cardiovascular comorbidities, for example [6].

However, in the absence of pre-existing neurological disorder, and when laboratory, imaging, and exam findings do not support a neurological picture, the determination of a structural versus primary etiology for LOBD becomes challenging. It has been posited that long-standing cerebrovascular insults secondary to atherosclerotic disease contribute to mania in old age, analogous to the concept of vascular depression [7]. Particularly, white-matter hyperintensities (WMH) can signify ischemic origin [8]. Zanetti et. al presented the case of a 72-year-old female with a history of hypertension and hyperlipidemia who displayed rapidly cycling mania and depressive symptoms and whose T2 and FLAIR MRI studies revealed diffuse hyperintensities in the subcortical and periventricular white matter [9].

WMH has, thus, been linked to the pathophysiology of LOBD, possibly via frontolimbic dysregulation [9-11]. Yet, in the absence of known atherosclerosis or metabolic syndrome, WMH can be viewed as coincidental findings, and the etiology could lie elsewhere (i.e., psychosocial stressors or incident events). Though research is sparse, a study with age-matched controls found that stressful life events likely predispose individuals to bipolar disorder, whether early or later in life [12].

Despite evidence showing 25% of cases of bipolar disorders occur in patients older than 60 years, there currently exist no formal treatment guidelines for LOBD [13,14]. A comprehensive narrative review on the evaluation and treatment of LOBD relied heavily on mixed-age population studies to draw inferences for management in older-age patients. Of note, lithium remains the mainstay monotherapy, especially in cases of minimal comorbid neurological disease, while electroconvulsive therapy (ECT) may play a therapeutic role in refractory cases as was conducted in this case. Recently, researchers reviewed both the behavioral variant of frontotemporal dementia and LOBD in order to solidify common and distinguishing features [15]. Ultimately, further research is needed into the underlying pathogenesis and etiology of LOBD for optimal diagnosis and treatment.

## Case presentation

Ms. S is a 79-year-old female who was admitted to our mental hospital for labile mood and physical aggression toward a police officer after being booked for criminal trespassing at a local store. She reportedly had been engaging in risky behaviors such as allowing strangers into her home, absent-mindedly leaving the stove on, and wandering at night. The family reports that the patient had been drinking large amounts of wine and smoking both marijuana and cigarettes, despite being a lifetime nonsmoker and nondrinker. Despite not finishing high school, she worked as a successful hair beautician and salon owner for 35 years. She lives by herself.

Upon admission, the patient appeared at the stated age with normal body habitus, poor eye contact, slightly disheveled appearance, and increased psychomotor activity. She was distractable and her speech was pressured. Her mood was elevated with a labile affect, alternating between periods of reactivity with verbal aggression and being tearfully apologetic. Her thought process was tangential, and her thought content consisted of grandiose delusions. She did not endorse hallucinations, suicidal ideation, or homicidal ideation. Insight and judgment were poor, and she was only oriented to herself. Physical examination was unremarkable.

Initial workup included complete blood count, comprehensive metabolic panel, lipid panel, hemoglobin A1c, thyroid stimulating hormone, rapid plasma reagin, urinalysis, and urine drug screen. Apart from low-density lipoprotein (LDL) being slightly elevated at 118 mg/dL and CO2 being mildly low at 18 mmol/L, all of the labs were negative and within normal limits. Tests for human immunodeficiency virus and hepatitis were negative. Folate was normal, but vitamin B12 was at the lower end of normal at 307 pg/mL; thus, she was started on an oral B12 supplement. CT of the brain revealed bilateral periventricular white matter with decreased attenuation, likely microangiopathic in origin, with chronic white-matter infarcts.

Ms. S has no significant medical conditions, takes no medications, and has no history of depression. Her sister has dementia of an unknown type; otherwise, there is no family history of neurologic or psychiatric disorders. Her son reports Ms. S was hospitalized twice prior for similar presentations, once in 2012 (at age 69) and once in 2022 just months prior to her current presentation. Prior to the 2012 episode, her husband passed, causing significant grief, and prior to both episodes, she reportedly had UTI and practiced fad diets. Both episodes were characterized largely by hypersexuality. Her current presentation is more severe as she did not display grandiosity or impulsivity previously. At baseline, her son reports that she is a private person who does not curse or use verbal aggression.

Her son reports that Ms. S had previously been diagnosed with “hypomania” and “bipolar mania” after 2012. However, she does not see any mental health providers. Consent to obtain collateral information from her primary care physician was obtained; her physician, who she regularly sees, reports no record of psychiatric history or medications.

Upon admission, the patient was started on valproic acid 500 mg twice daily as well as haloperidol 5 mg plus diphenhydramine 25mg nightly. The patient remained manic and labile with this regimen. Due to several incidents of physical aggression, she received intramuscular chlorpromazine, olanzapine, and lorazepam for agitation. These medications did not reduce her symptoms. On the eighth day of admission, Ms. S went to the emergency department for reports of somnolence and was found to have UTI, which was successfully treated with a course of antibiotics.

**Table 1:** Clinical progression of cognition and mania Progression of patient’s MoCA and YMRS scores out of 30 and 60, respectively, from baseline to successive sessions of ECT and at the time of discharge. MoCA: Montreal Cognitive Assessment, YMRS: Young Mania Rating Scale, ECT: electroconvulsive therapy

	Initial	Three ECT	Five ECT	Six ECT	Discharge
MoCA	11	17	16	19	24
YMRS	43	26	3	0	0

On the 15th day of admission after receiving consent from the patient’s son, Depakote was discontinued and she began ECT scheduled three times per week. To assess mental status, data points were obtained prior to and throughout the course of ECT as indicated by Table 1. Tools utilized were the Montreal Cognitive Assessment (MoCA) and the Young Mania Rating Scale (YMRS). The initial plan was to obtain data points at intervals of three ECT sessions. However, the patient responded very well to the first three sessions as noted by less pressured speech, less grandiose delusions, and cessation of physical and verbal aggression. Therefore, the frequency of ECT was decreased to two sessions per week, and data point collection dates were adjusted. After the fifth session, the patient was significantly more oriented and no longer manic, leading the team to stop ECT after six sessions.

As seen in Table 1, her MoCA and YMRS greatly improved from a baseline of 11 and 43, respectively, to scores of 24 and 0 on discharge. After the final ECT session, she remained calm with good eye contact, hygiene, and a normal rate of speech. She was euthymic with congruent affect. Her thought process was linear with no aberrations in thought content. She displayed good insight and judgment and was fully oriented. Ms. S was discharged on the 32nd day of admission upon the arrival of her family with valproic acid 500 mg BID and scheduled outpatient follow-up care with the diagnosis of bipolar I disorder.

## Discussion

Compared to other published reports, our case is unique in that Ms. S has no history of depression, no family history of psychiatric illness, and no comorbid medical conditions. While another case report does mention success with ECT, the patient had a positive family history, major depressive symptoms, and bipolar disorder which was characterized by rapid cycling [10]. Though Ms. S reportedly had two prior hospitalizations for less severe symptoms, she was not taking medications as confirmed by her primary care provider and pharmacy, and outside records were unable to be obtained. It is unclear if these episodes were mania/hypomania, or due to secondary causes, since she reportedly had UTIs prior to both these episodes. Furthermore, Ms. S suffered family losses prior to her first episode in 2012, leaving the possibility of complicated grief. A differential diagnosis of delirium was ruled out based on several considerations. First, her initial urinalysis on hospital day one was negative, and she presented with manic symptoms with no evidence of waxing and waning symptoms over the first week in the hospital until complaints of somnolence on day eight. Furthermore, there was no improvement in her manic symptoms after antibiotic treatment for over a week until she received several ECT treatments. It is also consistent with her diagnosis of “bipolar mania/hypomania” following the two previous psychiatric hospitalizations at 69 and 78.

The case of Ms. S highlights three important points. First, the case highlights the importance of a thorough workup to rule out secondary cases of mania. As documented by prior case reports, diagnosing bipolar I disorder in later life is predominantly a diagnosis of exclusion characterized by a thorough workup for organic causes such as brain tumors, dementia, metabolic disorders, cerebrovascular disease, and medication side effects. Though it has been hypothesized that late onset is of organic etiology, this cannot be substantiated because older populations tend to have a higher proportion of the aforementioned disorders; thus, one would expect higher rates of bipolar disorder onset in later life [16].

Second, the case emphasizes that the etiology of bipolar disorder, particularly late onset, remains poorly understood. There is ample evidence correlating WMH to LOBD [9-11]. However, their role has not been elucidated as not all patients with LOBD have WMH on imaging, and younger individuals with bipolar disorder also may have WMH [17].

Compared to controls, stressful life events were found to be more common in bipolar patients, regardless of age [12]. Life events, mostly unpleasant ones such as death, have been related to the first episodes of mania, while subsequent manic episodes require less stressful events to trigger mania [18]. Ms. S suffered three major family losses prior to her reported episode in 2012, indicating the possibility that her manic episode was triggered by those life events. Though there was no defining event prior to her current episode, Ms. S did report significant stress from caring for an ill family member and admitted prior to discharge that she thinks the stress caused her to “act funny.”

Third, the case highlights the need to revisit the treatment approach for LOBD. Treatment guidelines have yet to be established for older adults with bipolar disorder [13]. It is paramount to address this gap as LOBD have longer hospitalizations than those with early onset. While several articles have mentioned treatment approaches, drugs such as lithium, quetiapine, olanzapine, and valproate appear to be mainstays, while ECT is typically reserved for rapid cycling and refractory cases [19,20].

It is our belief that ECT should be considered earlier in patients with LOBD to not only decrease the burden of longer hospitalization but to return the patient to a euthymic state. We recognize that administering ECT does not negate the necessity of maintenance pharmacotherapy, as our patient was discharged with Depakote. Furthermore, the serial cognitive assessments MoCA and YMRS played a vital role in assessing our patient’s return to baseline. Applying this assessment to more patients while undergoing acute management of bipolar disorder may help reduce hospital stay time by using objective measures to track patients’ progress.

## Conclusions

The case of Ms. S not only underscores the known importance of a thorough workup to rule out secondary causes of mania but also represents a clarion call for revisiting and researching a comprehensive management approach to LOBD, for which serial cognitive assessments and ECT may play an important role. Lastly, our case emphasizes that the etiology of LOBD remains poorly understood, which may be reflected by the lack of recommended treatment approaches for these individuals.
